# Interaction of perfluorooctanoic acid with human serum albumin

**DOI:** 10.1186/1472-6807-9-31

**Published:** 2009-05-14

**Authors:** Ling-Ling Wu, Hong-Wen Gao, Nai-Yun Gao, Fang-Fang Chen, Ling Chen

**Affiliations:** 1 State Key Laboratory of Pollution Control and Resource Reuse, College of Environmental Science and Engineering, Tongji University, Shanghai 200092, PR China; 2 Key Laboratory of Yangtze River Water Environment of Ministry of Education, College of Environmental Science and Engineering, Tongji University, Shanghai 200092, PR China

## Abstract

**Background:**

Recently, perfluorooctanoic acid (PFOA) has become a significant issue in many aspects of environmental ecology, toxicology, pathology and life sciences because it may have serious effects on the endocrine, immune and nervous systems and can lead to embryonic deformities and other diseases. Human serum albumin (HSA) is the major protein component of blood plasma and is called a multifunctional plasma carrier protein because of its ability to bind an unusually broad spectrum of ligands.

**Results:**

The interaction of PFOA with HSA was investigated in the normal physiological condition by equilibrium dialysis, fluorospectrometry, isothermal titration calorimetry (ITC) and circular dichroism (CD). The non-covalent interaction is resulted from hydrogen bond, van der Waals force and hydrophobic stack. PFOA binding to HSA accorded with two-step binding model with the saturation binding numbers of PFOA, only 1 in the hydrophobic intracavity of HSA and 12 on the exposed outer surface. The interaction of PFOA with HSA is spontaneous and results in change of HSA conformation. The possible binding sites were speculated.

**Conclusion:**

The present work suggested a characterization method for the intermolecular weak interaction. It is potentially useful for elucidating the toxigenicity of perfluorochemicals when combined with biomolecular function effect, transmembrane transport, toxicological testing and the other experiments.

## Background

It is well known that the Teflon event involving the Dupont Company of USA drew serious international attention to perfluorooctanoic acid (PFOA) [1]; PFOA is formed from the raw materials used in the production of Teflon-lined non-stick cooking appliances. Fluoropolymers such as Teflon have very good performances e.g. as fire retardants and for oil and fat resistance; their byproducts such as PFOA can be formed by cooking, burning and environmental degradation. PFOA is still widely used in basic processes in the aviation, automobile, building materials, chemicals, electronic, semiconductor and textile industries. It is persistent and non-biodegradable and becomes widely distributed in nature, e.g. water [2], biological bodies [3], human tissues [4] and foods [5]. It can certainly enter the gastrointestinal tract via the intake of foods and water and then it is absorbed and permeates into the blood and various tissues. Sampling studies have revealed the presence of PFOA in the bloods of over 90% of US residents [6]. It may have serious effects on the endocrine, immune and nervous systems and it can be delivered to the fetus through the umbilical cord and can accumulate [7]. It can also cause cancers of the liver, testis, pancreatic and mammary glands [8], and can lead to embryonic deformities and other diseases [9,10]. In recent years, PFOA has become a significant issue in many aspects of environmental ecology, toxicology, pathology and life sciences [11,12].

Human serum albumin (HSA) is a major protein component of blood plasma but is also found in the interstitial fluid of body tissues. In mammals, albumin is synthesized by the liver and has a half-life of 19 days in the circulation [13]. It is the major contributor to the oncotic pressure of the blood plasma [14]. It is called a multifunctional plasma carrier protein because of its ability to bind an unusually broad spectrum of ligands e.g. inorganic ions, various drugs, amino acids, fatty acids, etc. Binding to HSA facilitates their transport throughout the circulation [15]. Without doubt, interaction of any toxicant with HSA influences the transport of nutrients and drugs. Recently, studies have been conducted on the binding of organic contaminants or toxins to HSA e.g. arazine [16], ochratoxin [17], methyl parathion [18] and arsenic [19]. Bindings of PFOA to biomacromolecular such as rat and Human plasma proteins [20], rat liver-form and kidney-form alpha 2u-globulins [21], have been investigated at room temperature. The interaction of organic contaminants and HSA is always affected by various environmental conditions such as pH, strength and temperature [22]. In this work, we investigated the interaction of PFOA with HSA by equilibrium dialysis, fluorospectrometry, isothermal titration calorimetry (ITC) and circular dichroism (CD) under normal physiological condition, pH 7.40 and 0.15 M electrolyte and 37°C. The object is to analyze the interaction forces, sites and type and then further understand the toxigenicity of PFOA.

## Results and discussion

### Equilibrium dialysis of PFOA

**Figure 1:**
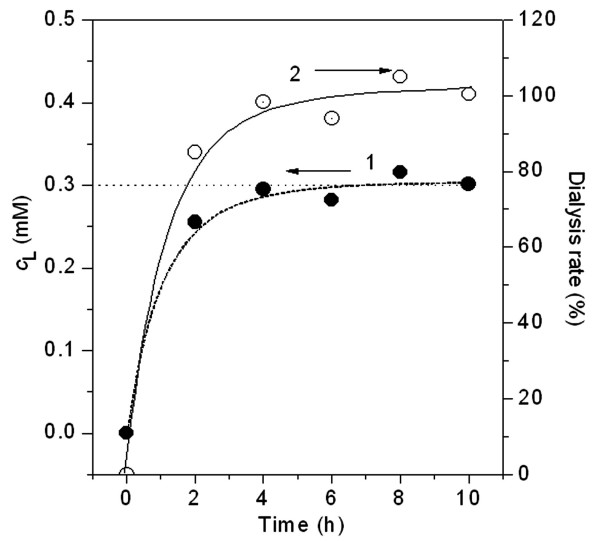
**Equilibrium dialysis of PFOA: (1) – Change of PFOA concentration (c_L_) in the dialysis solution initially containing 9.0 ml of pH 7.40 BR buffer and 27.0 ml of distilled water, while 3.0 ml of pH 7.40 BR buffer, 3.0 ml of 5.00 Mm PFOA and 4.0 ml of distilled water were mixed in the bag at the beginning of the dialysis**. PFOA in the dialysis solution was determined at various dialysis times. (2) – Variation of the PFOA dialysis rate calculated by (initial molarity of PFOA in bag – 4c_L_)/initial molarity of PFOA in dialysis membrane, which was used to estimate the dialysis equilibrium time.

The equilibrium dialysis method was used to investigate the interaction of PFOA with HSA. The PFOA concentration in the dialysis solution was measured at 37°C at various dialysis times with no protein in the membrane. The PFOA dialysis rate approaches 90% at 2 h and exceeds 98% after 4 h (Fig. 1). PFOA passes freely through the semi-permeable membrane until equilibrium reached. HSA solution was put into the dialysis membrane as dialysate instead of PFOA, but it was found only in the dialysis bag. Therefore, the equilibrium dialysis is suitable for investigating the interaction of PFOA with HSA. The dialysis solution was sampled and measured spectrophotometrically to determine the PFOA concentration after more than 4 h of dialysis.

### Characterization of the interactions of PFOA with HSA

The interaction of PFOA with HSA is summarized as follows:



Both *c*_L0 _and *c*_M0 _are the initial mole concentrations of PFOA and HSA, *c*_L _is the equilibrium concentration of PFOA described above. *N *is the saturation binding number of PFOA. The effective fraction (*f*) of PFOA bound to HSA and its molar binding ratio (γ) are calculated by the relations: *f *= 1-*c*_L_/*c*_L0 _and γ = *fc*_L0_/*c*_M0_. The γ value will approach *N *with increasing PFOA.

**Figure 2:**
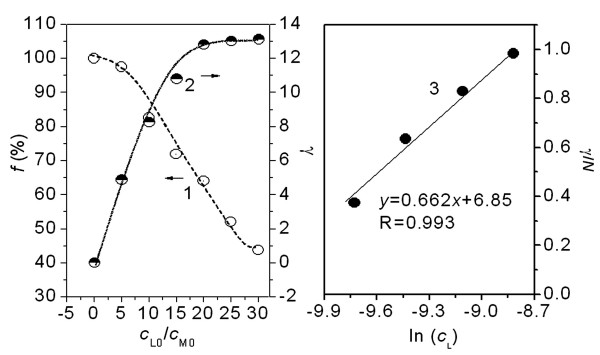
**Effect of PFOA (initial concentration from 0 to 1.75 mM in the dialysate) on binding to HSA, initially 0.033 mM**. (1) and (2) – *f *and γ of PFOA; (3) – plots γ/N vs. *ln*(c_L_). All the solutions were in 0.15 M NaCl at pH 7.40 at 37°C

In fact, pH varies widely among normal tissues in the human body. For example, less than pH 3.0 in gastric fluid, pH 6.0 in liver and saliva, pH 4.0 – 5.0 on the skin and around pH 7.40 in blood and intestinal tract. Normal human temperature is 37°C and the electrolyte concentration is between 0.8 and 0.9% (approximately 0.15 M). In this present work, the experiments were conducted in 0.15 M NaCl at pH 7.40 at 37°C. By measuring a series of PFOA solutions containing known concentrations of HSA at pH 7.40, the *f *and γ values were calculated according to the above equations. The *f *decreases linearly with increasing PFOA concentration from curve 1 in Fig. 2, but γ increases from curve 2. The binding of PFOA approaches to a constant maximum at 13 when *c*_L0_/*c*_M0 _is more than 20 from curve 2. The number of amino acid residues positively charged e.g. Lys, His and Arg is 98 in HSA, which is no correlation with such an *N *value. Different from a sulfonic azo ligand [23], the PFOA binding to HSA doesn't results from ion-pair attraction. Strong intermolecular forces e.g. hydrogen bond, van der Waals forces and hydrophobic interaction may be involved. The Temkin isothermal model,  (*K*_a _– adsorption constant, M^-1^, Δ*Q *– saturation adsorption energy, *T *– the Kelvin temperature and *R *– the gas constant, 8.314 J mol^-1^K^-1^) was used to fit the above experimental data. From curve 3 (Fig. 2), the interaction of PFOA with HSA corresponds to chemical monolayer adsorption. Both Δ*Q *and *K*_a _were calculated to be -3.89 kJ/mol and 3.12 × 10^4 ^M^-1^. The adsorption of PFOA on HSA is exothermic and non-covalent [24], i.e. no strong bond was formed between PFOA and HSA.

**Figure 3:**
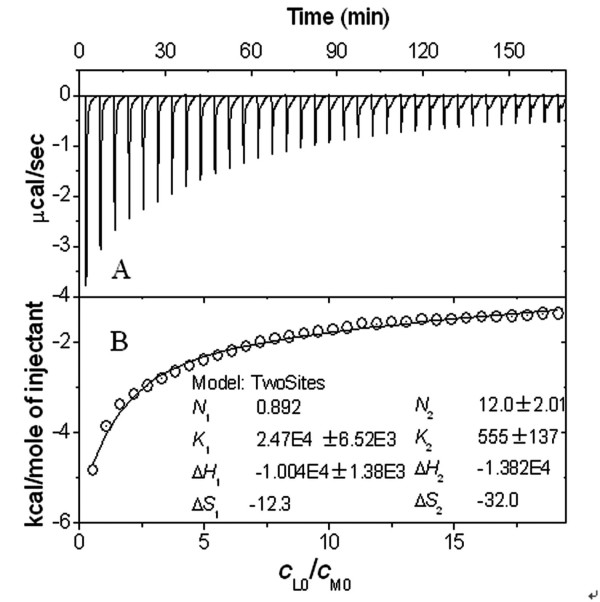
**(A) ITC titration profile of PFOA-HSA binding at pH 7.40**. The temperature was 37°C and all the solutions contained 0.15 M NaCl. Each pulse corresponds to a 6.0-μl injection of 2.50 mM PFOA into the ITC cell (1.4685 ml) containing 0.010 mM HSA. (B) The area of each peak in A was integrated and corrected for the heat of dilution, which was estimated in a separate experiment by injecting the PFOA into the buffer.

In order to understand the mechanism of PFOA-HSA binding, some detailed thermodynamic data are needed. The ITC measurement may provide information on thermodynamic quantities such as enthalpy and heat capacity changes during the molecular interaction directly from the heat produced by the reaction, and have been used to study, for example, protein interactions [25], DNA triplex formation [26] and HIV protease activity [27]. Fig. 3A depicts a typical isothermal titration profile obtained by injecting PFOA into the ITC cell containing HSA. The resulting values were plotted as a function of *c*_L0_/*c*_M0 _and fitted to a two-step sequential binding model by a nonlinear least squares method (Fig. 3B). Values for the equilibrium constant (*K*_*b*_), enthalpy change (Δ*H*) and entropy change (Δ*S*) of the PFOA-HSA reaction were obtained and calculated by the Gibbs free energy (Δ*G*) equation: Δ*G *= -*RTlnK*_b _= Δ*H*-*T*Δ S. The thermodynamic parameters derived from this curve are summarized in Table 1. Because both Δ*H*_*i *_values are much less than 60 kcal/mol [24], the non-covalent binding of PFOA to HSA is confirmed.

**Table 1:** Thermodynamic property of the HSA-PFOA interaction at pH 7.40 at 37°C

*i*-th step	*c*_L0_/*c*_M0_	*N*_*i*_	*K*_*b*, *i*_ (10^3 ^M^-1^)	Δ*H*_*i*_, (kcal/mol)	ΔS_i_ (cal/(mol·K))	Δ*G*_*i*_ (kcal/mol)
1	< 1.5	1	24.7 ± 6.5	-10.0	-12.3	-6.19
2	1.5–20	12	0.55 ± 0.14	-13.8	-32.0	-3.88

**Figure 4:**
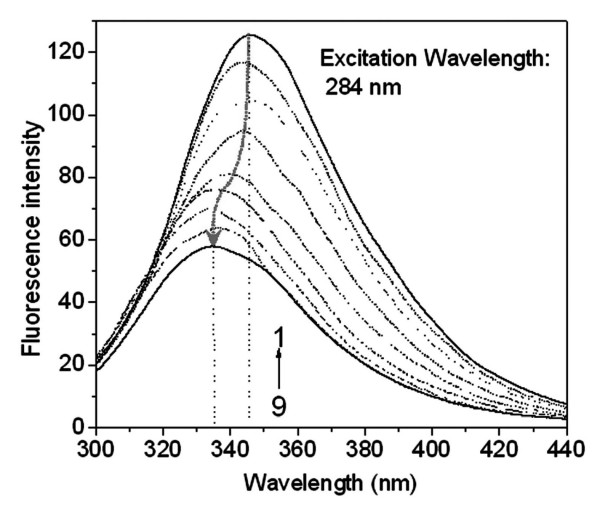
**Variation of the intrinsic fluorescence of HSA (c_M0 _= 0.010 mM) with increase of PFOA (c_L0 _= 0 – 0.300 mM) at pH 7.40**. From (1) to (9): c_L0_/c_M0 _is 0, 1.0, 1.5, 2.0, 3.0, 5.0, 10.0, 20.0 and 30.0.

**Figure 5:**
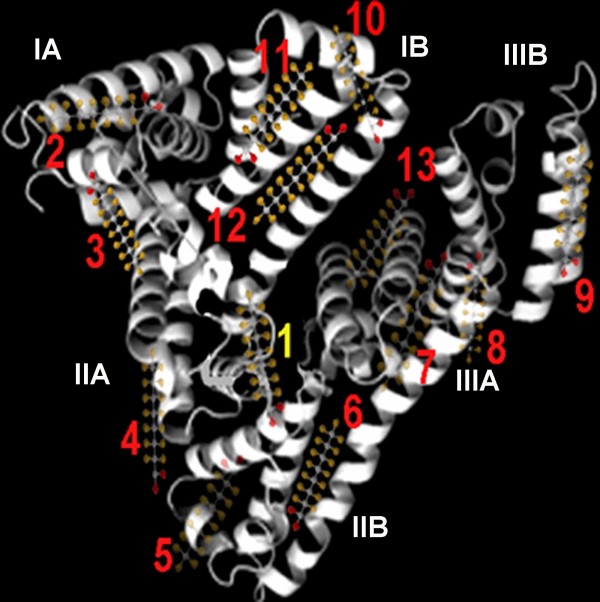
**Cartoon illustrating the binding of PFOA to HSA**. (1) – possible PFOA binding site in the 1st step; from (2) to (13) – PFOA binding sites in the 2nd step.

PFOA is more lipophilic in aqueous solution and it decreased obviously the aqueous surface intensity. Fifteen electrophilic F-groups in PFOA can attract strongly the lone pair electrons of polar side groups of peptide chain, e.g. F⋯N and F⋯O halogen bonding [28]. HSA is consisted of three homologous all α-helical domains (I–III), each divided into two subdomains [14]. All the helixic subdomains are distributed round a hydrophobic intracavity and the hydrophilic side groups of HSA exposed on the outer surface. In the first step, only one PFOA molecule bound on HSA (Table 1). The *f *of PFOA is always more than 90% from curve 1 in Fig. 2 when the *c*_L0_/*c*_M0 _is less than 5. Therefore, the first step is complete at approximately 1.5 of *c*_L0_/*c*_M0_. HSA binding PFOA caused the entropy decreasing (Δ*S*_1 _< 0) in this step. The refolding of HSA occurred by resuming the released heat. From Δ*G*_1 _value, the PFOA-HSA reaction is spontaneous. Moreover, the higher *K*_b, 1 _value indicated that the binding of the first PFOA molecule to HSA is firmer. From curve 1 to 9 in Fig. 4, the blue shift of peak emission wavelength from 345 to 335 nm indicated that the static quench of the HSA's intrinsic fluorescence occurred in the presence of PFOA, specially obviously when *c*_L0_/*c*_M0 _is less than 2. Thus, the first PFOA molecule may bind to the side group of Tyr. As a result, the deformable linear PFOA may insert into the hydrophobic intracavity of HSA and bridge between subdomains IIA and IIB (No. 1 in Fig. 5) across Trp 213 by F⋯N and F⋯O halogen bonding with the polar side groups of Asn, Gln, Asp, Glu, Arg, His, Tyr and Trp and hydrophobic interaction with the non-polar side groups, e.g. Val, Leu, Met, Ala and Ile. The first step binding of PFOA to HSA is similar to that of long chain fatty acids to HSA [14].

Besides a long carbon chain, PFOA has strong extensibility on interface of water – particles so that it may spread on the exposed outer surface of HSA. When *c*_L0_/*c*_M0 _is more than 2, PFOA began to bind on the hydrophilic surface of HSA till a saturation (*N*_2 _= 12) via the polar bonds e.g. ionic interaction, hydrogen bond and F⋯N and F⋯O halogen bonding (Fig. 3B). By comparison of *N *obtained by equilibrium dialysis (Fig. 2) and that (*N *= *N*_1 _+ *N*_2 _= 13) obtained by ITC (Fig. 3B), two methods achieves the same result. From the higher Δ*H*_2 _value, the binding sites of PFOA may bridge between any two helixes all over the outer surface of HSA (Fig. 5). From Δ*G*_2 _value (Table 1), the PFOA-HSA reaction is spontaneous. In the 2nd step, the binding of PFOA to HSA caused an obvious entropy decreasing, i.e. more negative Δ*S*_2 _(Table 1), the HSA structure changed refolding.

**Figure 6:**
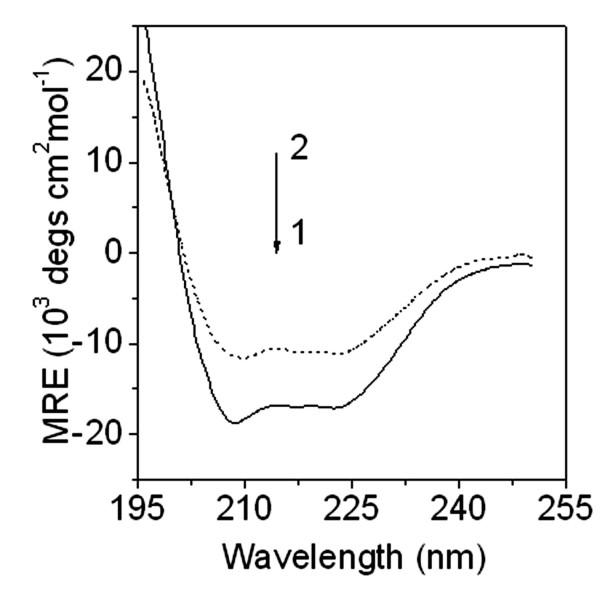
**Molar ellipiticity curves for HSA (0.010 mM) solutions at pH 7.40**. (1) – no PFOA and (2) – 1.0 mM PFOA.

By comparison of the emission spectra in Fig. 4, the obvious blue shift of peak wavelength was found when *c*_L0_/*c*_M0 _is more than 2. This indicates an obvious change of HSA conformation, too. As a result, the interaction of PFOA with HSA is assignable under normal physiological condition. In addition, the β-pleated sheet content of HSA decreased obviously and its α-helix increased by 15% in the presence of PFOA from the change of CD spectra of HSA (Fig. 6). Thus, the second step in PFOA binding changed a substantial part of the β-pleated sheet of HSA into α-helix form. Without doubt, such a binding would cause a great change of HSA structure and affect the function of HSA in blood.

## Conclusion

The current work investigated the interaction of PFOA with HSA in the normal physiological acidities of blood and intestinal tract tissue where PFOA molecules may be present. The interaction of PFOA with HSA accorded with the Langmuir isothermal model in two-step sequence, in which only one PFOA molecule entered the hydrophobic intracavity in the first step and 12 PFOA molecules binding on the hydrophilic outer surface in the second step. The interaction of PFOA and HSA is spontaneous and the non-covalent bond results in change of HSA conformation. The possible binding sites were also speculated. The present work proposed a determination and characterization method for the intermolecular weak interaction. If combined further with the other experiments e.g. biomolecular function effect [29], cell membrane transport of contaminant [30] and toxicological testing [31], it is more helpful for elucidating the toxigenicity of perfluorochemicals.

## Methods

### Instruments and chemicals

Model S-4100 spectrophotometer (Sinco Co., Korea), which was computer-controlled by Labpro Plus firmware (Version 060105); Model MSC-ITC system (MicroCal Inc., USA) with measurement software; Model J-715 CD Spectropolarimeter (Jasco Instrum., Japan) with secondary structure Estimation-Standard Analysis Measurement software (715/#B014460524, JASCO); Model RC 30 – 5K semi-permeable membrane (Molecular Weight Cut Off 5 KDa, Shanghai Green Bird STD). Both 0.100 mM HSA (Sigma) was prepared and stored at 4°C. The other solutions were prepared: a standard PFOA solution (New Jersey, USA) (5.00 mM); Britton-Robinson (BR) buffers (pH 7.40); ECR (1.00 mM, A. R., Sigma) solution; CPC (1.00 mM, purity > 99%, Shanghai Reagents).

### Determination of PFOA

A simple and rapid spectrophotometry method for determining PFOA was developed. The anionic color ligand eriochrome cyanine R (ECR) and cationic surfactants cetylpyridinium chloride (CPC) were employed to determine PFOA. The detailed analytical procedure was described by [32].

### Equilibrium dialysis

**Figure 7:**
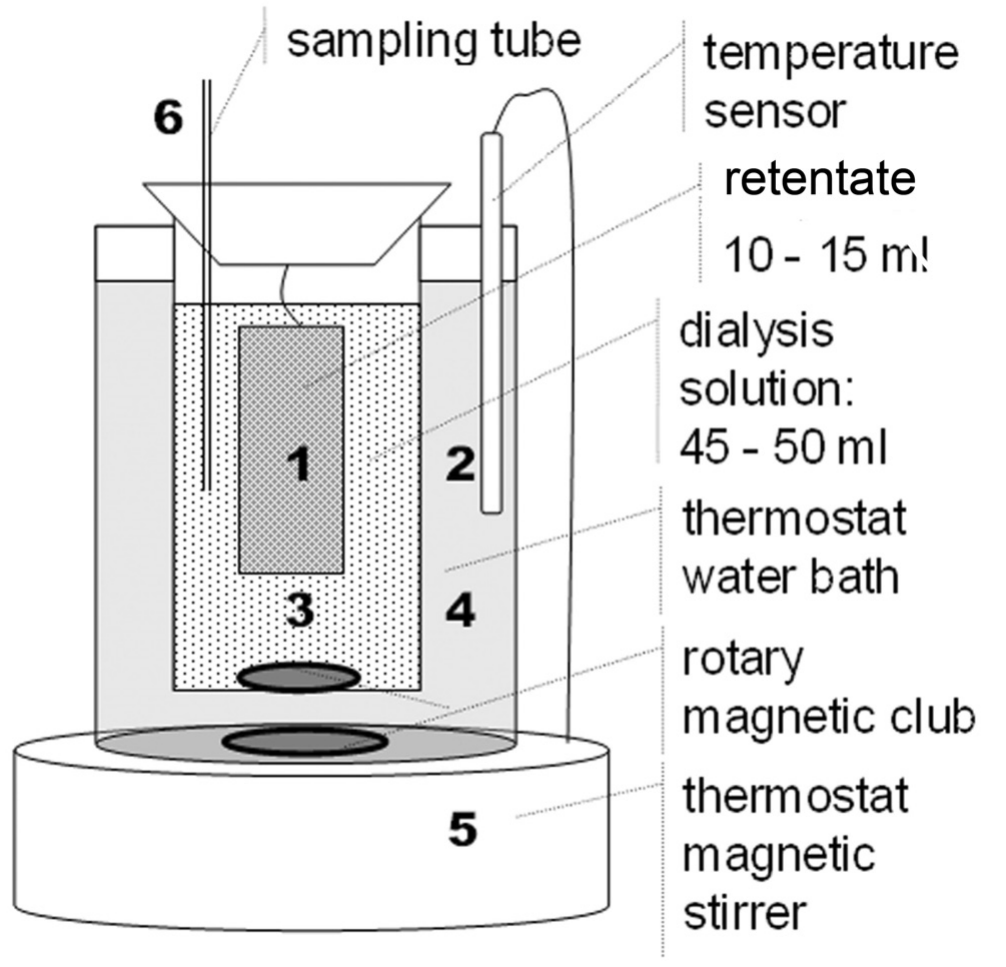
**The new dialyzer was designed as illustrated: (1) – semi-permeable membrane with 15.0 ml of retentate; (2) – the temperature sensor for maintaining the reaction at constant 37°C (3) – Dialysis solution less than 50.0 ml; (4) – water bath at constant 37°C**. The apparatus was placed on a thermostated magnetic stirrer (5) and rotary magnets were used to mix solutions 3 and 4 thoroughly. The PFOA concentration in solution (3) was determined from the sampling tube (6).

Unlike a color ligand [23], PFOA does not absorb visible light strongly so the interaction cannot be characterized by VIS spectrophotometry. Therefore, equilibrium dialysis was used. We designed a new dialysis device (Fig. 7), where 12.0 ml solutions containing 3.0 ml of BR buffer (pH 7.40), 0.15 M NaCl, 0.033 mM HSA (*c*_M0_), a series of PFOA concentrations (*c*_L0_) and distilled water were mixed and transferred to dialysis bags (1). A solution (36.0 ml) containing 0.15 M NaCl, 9.0 ml of BR buffer and distilled water was added to the dialysis cup (3). The constant temperature (2) of the water bath (4) was kept at 37°C by adjusting the thermostat magnetic stirrer (5). After 4 h, 1.0 ml of the dialysis solution (3) was collected with the sampling tube (6) and the PFOA concentration (*c*_L_) was determined by spectrophotometry.

### ITC measurement

By means of a ITC device, PFOA solution (2.50 mM in pH 7.40 BR buffer) was injected about 40 times in 6-μl increments at 3-min intervals into an isothermal cell containing HSA (10.0 μM in pH 7.40 BR buffer). The cell temperature was maintained at 37°C and all the solutions contained 0.15 M NaCl. Heats of dilution of PFOA, obtained separately by injecting into the buffer, were used to correct the data. The corrected heats were divided by the number of moles injected and analyzed using the Origin software (version 7.0).

### CD measurement

BR buffer (1.0 ml, pH 7.40) was mixed with 0.010 mM HSA in three flasks; or 1.00 mM PFOA was added and the solutions were diluted to 10.0 ml with distilled water. Simultaneously, a reagent blank without PFOA was prepared. Before measurement, all the solutions were diluted from 0.1 to 10.0 ml with 10% BR buffer. Each sample was allowed to equilibrate for 15 min, then injected into a 0.1-cm light path cell, and the mean residue ellipticity (MRE) of HSA was measured between 195 and 250 nm by spectropolarimetry.

## Authors' contributions

LLW carried out all of the experimental operations except ITC measurement. HWG designed research and wrote the paper. NYG treated the data. FFC performed ITC measurement. LC provided the detection method. All authors read and approved the final manuscript.
